# The complete mitochondrial genome of the *Rhacophorus chenfui* Liu, 1945 and its phylogenetic analyses

**DOI:** 10.1080/23802359.2024.2427829

**Published:** 2024-11-12

**Authors:** Lichun Jiang, Simin Chen, Weibo Kong, Wanqing Song, Jingfeng Liu, Chunxiu Liu, Peng Liu

**Affiliations:** a Key Laboratory for Molecular Biology and Biopharmaceutics, School of Life Science and Technology, Mianyang Normal University, Mianyang, Sichuan, P.R. China; b Ecological Security and Protection Key Laboratory of Sichuan Province, Mianyang Normal University, Mianyang, Sichuan, P.R. China

**Keywords:** *Rhacophorus chenfui*, Rhacophoridae, mitogenome, characterization, phylogenetic analysis

## Abstract

We described the complete mitogenome sequence of *Rhacophorus chenfui* in this research. The circular mitogenome of R. *chenfui* is total length with 20,520 bp, encoded 39 genes (24 tRNA genes, 13 protein-coding genes, 2 rRNA genes) and two D-loop regions. The base composition of the mitogenome is 33.46% A, 30.80% T, 23.05% C, and 12.69% G. All tRNAs have the typical clover-leaf structure except for tRNA^Ser1(AGN)^ which have a reduced DHU arm. The results showed that R. *chenfui* is closely related with R. *schlegelii*, R. *arboreus*, *Zhangixalus omeimontis* and *Z. dugritei*. This work enriches the library of Rhacophoridae mitoenomes and provides a valuable resource for understanding the evolutionary history of *Rhacophorus*.

## 1. Introduction

The Chinese whipping frog, *Rhacophorus chenfui* (Liu, 1945) belong to the family Rhacophoridae, genus *Rhacophorus*. The genus *Rhacophorus* includes approximately 80 species distributed throughout Asia (Rowley et al. 2010; Frost 2020). *R. chenfui* is endemic to China, and occurs in central and southeastern Sichuan, extreme northern Yunnan through northern Guizhou and southern Chongqing, into northwestern Hunan and southwestern Hubei. Isolated populations have been also reported from northwestern Fujian, southwestern Jiangxi, and northeastern Sichuan (Frost 2020). It probably occurs more widely than current records suggest, especially in areas between known sites. This species has been recorded from an elevational range of 900–3,000 m asl (Fei et al. 2012). It inhabits creeks, ponds, paddy fields, small ditches, and the surrounding habitats (such as lowland and montane shrublands and forests) in hilly areas (Fei et al. 2012). In the past decade, *R. chenfui* has been studied widely in behavioral ecology aspects, but it is reported rarely about its mitochondrial genome. Mitochondrial DNA has played an important role as molecular marker in studying population genetic structure and evolutionary history of species (Wang et al. 2018; Xue et al. 2016; Zhang et al. 2018). In the study, we sequenced and determined the complete mitochondrial genome of *R. chenfui*, and its taxonomic status in Rhacophoridae species was analyzed, which will help us improve our understanding of the mitogenome features of genus *Rhacophorus*, and evolutionary relationships of the family Rhacophoridae.

## 2. Materials and methods

### 2.1. Sample collection

**Figure 1.**
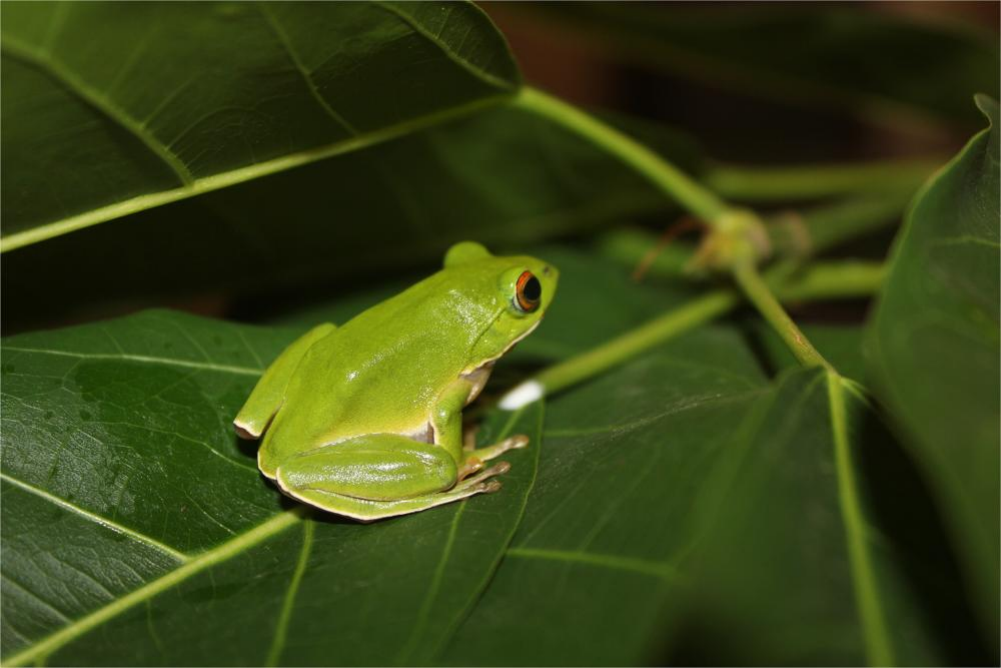
The specimen of *Rhacophorus chenfui* from the Wawushan Town, Hongya County, Sichuan Province, China (photo by Lichun Jiang).

This specimens *R. chenfui* (Figure 1) used in this study was sampled from the Wawushan Town, Hongya County, Sichuan Province, China in July 2019 (103°58′3.05″E, 29°41′1.40″N). The collected specimen underwent morphological identification (AmphibiaChina 2023; IUCN 2023) and was subsequently preserved in 95% ethanol. The distribution of this species is shown in Figure S1. After *R. chenfui* were captured in the wild, first the flippers between their toes were disinfected with alcohol. Approximately 30 milligrams of flipper tissue was cut off, and then the area was disinfected again before the animals were put back into the wild. Subsequently, the flipper samples were preserved in 95% ethanol and deposited at the Ecological Security and Protection Key Laboratory of Sichuan Province under the voucher number JL2019070916 (http://zdsys.mnu.cn/; person in charge of the collection: Lichun Jiang; email: jiang_lichun@126.com).

### 2.2. Methods

Genomic DNA was extracted from the webs of the frog toe using the TIANamp Animal Genomic DNA Kit (TIANGEN, Beijing) following the operation instructions. To detect conserved sequences, we aligned mitochondrial sequences from multiple species within the genera *Zhangixalus* and *Rhacophorus* using MEGA7 (Kumar et al. 2016). Partial PCR primers were designed based on the alignments of the relatively conserved regions. Another part of the primers derived from the literature (Kurabayashi and Sumida 2009). Then, PCR amplification was performed using the designed 12 pairs of gene primers (Table 1). The PCR amplification fragments were separated by 0.9% agarose gel electrophoresis and the fragments were scanned with a gel imager (Figure S2). Each purified amplification products were subjected to automated bidirectional sequencing using an ABI 3730 sequencer based on Sanger sequencing method. DNA Baser software (http://www.DNABaser.com) was used to assembly the whole mitogenome sequence based on the overlapping portions (200–350 bp in the overlapping region) of the sequencing products. The annotation process was executed using MitoMaker (Bernt et al. 2013). Subsequently, we manually corrected each annotated gene fragment. The mitogenome sequence and gene annotations were submitted to the National Center for Biotechnology Information GenBank database under the accession number MN248535. The circular genome map was generated using the OGDRAW version 1.3.1 (Greiner et al. 2019).

**Table 1.** PCR Primers for the *Rhacophorus chenfui* mitochondrial genome.

No.	Primer name	Sequence 5′–3′	Primer length (bp)	References
1	JF1	ATTAAGATAAAGCCCTTCTAGAA	23	This study
	JR1	AATACCATTGGTGTCCCACG	20	
2	JF2	CAAGAYGCRRYHTCHCCNATYATAGAAGA	29	Kurabayashi and Sumida 2009
	JR2	CCTTCWCGRAYNAYRTCTCGYCAYCAYTG	29	
3	JF3	GCMCACCAAGCWCAYGCHTWYCAYATRGT	29	Kurabayashi and Sumida 2009
	JR3	GADCCDGCRATDGGDGCYTCDACRTG	26	
4	JF4	CACTACGCAGCAGACACCTC	20	This study
	JR4	CAAGGGAAGGTCCTATCAAGT	21	
5	JF5	ATGGTGGTATAATAGTATGGTGT	23	This study
	JR5	GTTGTTGGGAATAAGGGTGT	20	
6	JF6	ACCTCATACGCAAACTCAGC	20	This study
	JR6	TACCATCATTTTAATAGGTGGA	22	
7	JF7	CCCACATGTATAATTAACAGATT	23	This study
	JR7	GGAGTAATCTTTCGTTTTGTAT	22	
8	JF8	TTCGCAAAGCAAATACCCACA	21	This study
	JR8	CGCCGACTAATATCAATTTG	20	
9	JF9	CCACACCYHCAAGGGHAYTCAGCAGT	26	Kurabayashi and Sumida 2009
	JR9	CTTYGCACGGTYAGRRTACCGCGGCCGT	28	
10	JF10	CCCGCCTGTTTACCAAAAACAT	22	Kurabayashi and Sumida 2009
	JR10	ACRTTRAANCCNGANACHAGTTCWGAYTC	29	
11	JF11	CGRGCHGTHGCHCAAACNATYTCHTAYGA	29	Kurabayashi and Sumida 2009
	JR11	AAGCTCKCTGGAWWGAGYGTTTAGCTGTTAA	31	
12	JF12	GTCGCCCAAACAATCTCATATGA	23	Kurabayashi and Sumida 2009
	JR12	AGGAGGGCTTTATCTTAAT	19	

To examine the phylogenetic position of the genera *Zhangixalus*, *Rhacophorus*, *Polypedates*, *Buergeria* and some related taxa, 42 complete mitogenome sequences were selected as ingroups, and 2 species in Microhylidae served as outgroups. The combined amino acid sequences, which were integrated by combining 13 protein-coding genes (PCGs) from each species, were used to construct the phylogenetic tree. These combined sequences were then submitted to perform multiple alignment using ClustalW, a built-in tool in MEGA v11 software (Tamura et al. 2021; Stecher et al. 2020). Preliminary trees for the heuristic search were automatically derived by applying the Neighbor-Joining and BioNJ algorithms to a pairwise distance matrix estimated utilizing the Jones-Taylor-Thornton (JTT) model. The topological arrangement with the highest log likelihood value was subsequently chosen. Additionally, the evolutionary history was elucidated through the Maximum Likelihood (ML) method, employing the JTT matrix-based model proposed by Jones et al. (1992). Finally, an online tool, iTOL, was used to visualize the resulting tree.

## 3. Results

### 3.1. Mitogenome organization

**Figure 2.**
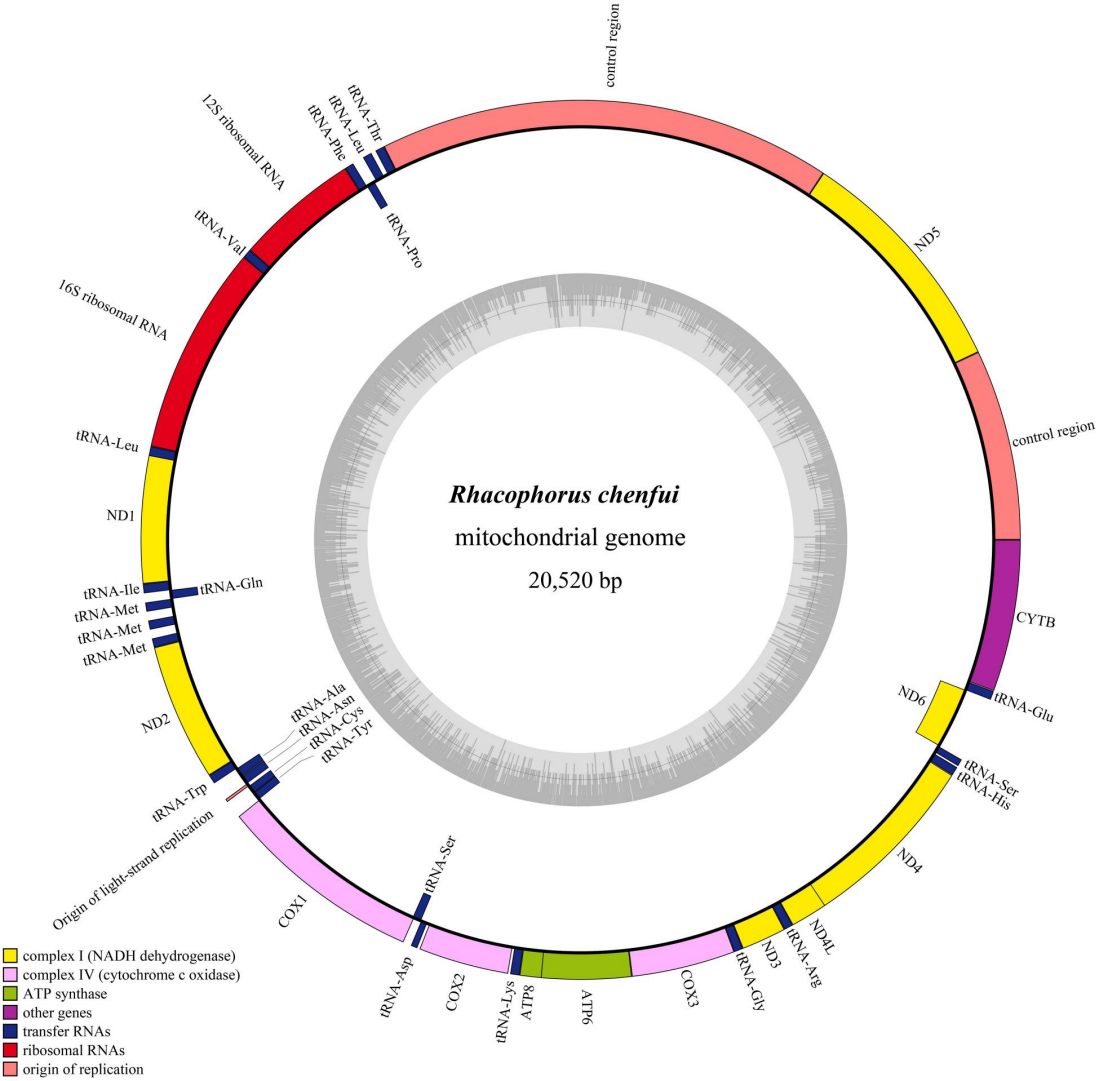
Complete mitochondrial genome organization and gene arrangement of *Rhacophorus chenfui*. Genes coded on the H strand are directed to the outer ring (clockwise direction), while the genes coded on the L strand are indicated in the interior of the ring (anticlockwise direction). Genes are abbreviated as follows: ATP6 and ATP8 (subunits 6 and 8 of ATPase), COXI-COXIII (cytochrome c oxidase subunits 1–3), cytb (cytochrome b), ND1-ND6 and ND4L (NADH dehydrogenase subunits 1–6 and 4 L), 12S rRNA and 16S rRNA (ribosomal RNA of 12S and 16S), CR (control region). One-letter amino acid abbreviations were used to label the corresponding tRNA genes.

The complete circular mitochondrial genome of *R. chenfui* is 20,520 bp in length (Figure 2), showing a base composition of: 33.46% A, 30.80% T, 23.05% C, and 12.69% G, with a slight bias toward A + T, which is similar to other Sparidae species (Zhang et al. 2018; Pyron and Wiens 2011). Its mitogenome contains two rRNA genes, 13 PCGs, 24 tRNA genes, and two non-coding control regions, all of which are similar in length to their counterpart genes in amphibians (Sano et al. 2005). Among the 39 genes, except for *ND6* gene and eight tRNA genes (*tRNA^Pro^*, *tRNA^Gln^*, *tRNA^Ala^*, *tRNA^Asn^*, *tRNA^Cys^*, *tRNA^Tyr^*, *tRNA^Ser^* and *tRNA^Glu^*), which are encoded on the light strand, the remaining genes are encoded on the heavy strand (Figure 2). For 13 PCGs, *ND2* uses ATT as start codon, *COXI* uses ATA as start codon, and codon of 11 PCGs (*ND1*, *ND3*, *ND4*, *ND4L*, *ND5*, *ND6*, *COXII*, *COXIII*, *ATP6*, *ATP8* and *CYTB*) are started with ATG. The stop codon of PCG is generally TAA/TAG or incomplete. In the study, the six PCGs (*ND2*, *COXII*, *ATP8*, *ND5*, *ND4L* and *CYTB*) ended with TAA as stop codon. But the stop codon of *COXI* is AGG, the stop codon of *ND6* is AGA. The rest of five genes (*ATP6*, *COXIII*, *ND1*, *ND3* and *ND4*) are found to be incomplete T– stop codon, which may be presumably completed by posttranscriptional polyadenylation with poly A tail (Boore 2001; Ojala et al. 1981). The 24 tRNA genes are interspersed along the whole genome, and ranging in size from 56 bp (*tRNA^Ser^*) to 73 bp (*tRNA^Asn^*_,_ *tRNA^Leu^*). Among the 2 rRNA genes, 12S rRNA is located between *tRNA^Phe^* and *tRNA^Val^* with 936 bp length, 16S rRNA is located between *tRNA^Val^* and *tRNA^Leu^* with 1,580 bp length. The mitochondrial sequence contains two D-loop regions (lengths of 3,104 bp and 1,304 bp), which are located between *ND5* and *tRNA^Thr^*, *CYTB* and *ND5*, respectively. The phenomen of 2 control regions is quite common among Rhacophoridae species (Rowley et al. 2010; Sano et al. 2005). Moreover, a putative origin of the Light-strand replication (O_L_) as the small non-coding region, is located between *tRNA^Asn^* and *tRNA^Cys^* within WANCY tRNA cluster with the length of 25 bp, it is similar to most vertebrate mitogenomes (Shan et al. 2016).

### 3.2. Phylogenetic analysis

**Figure 3.**
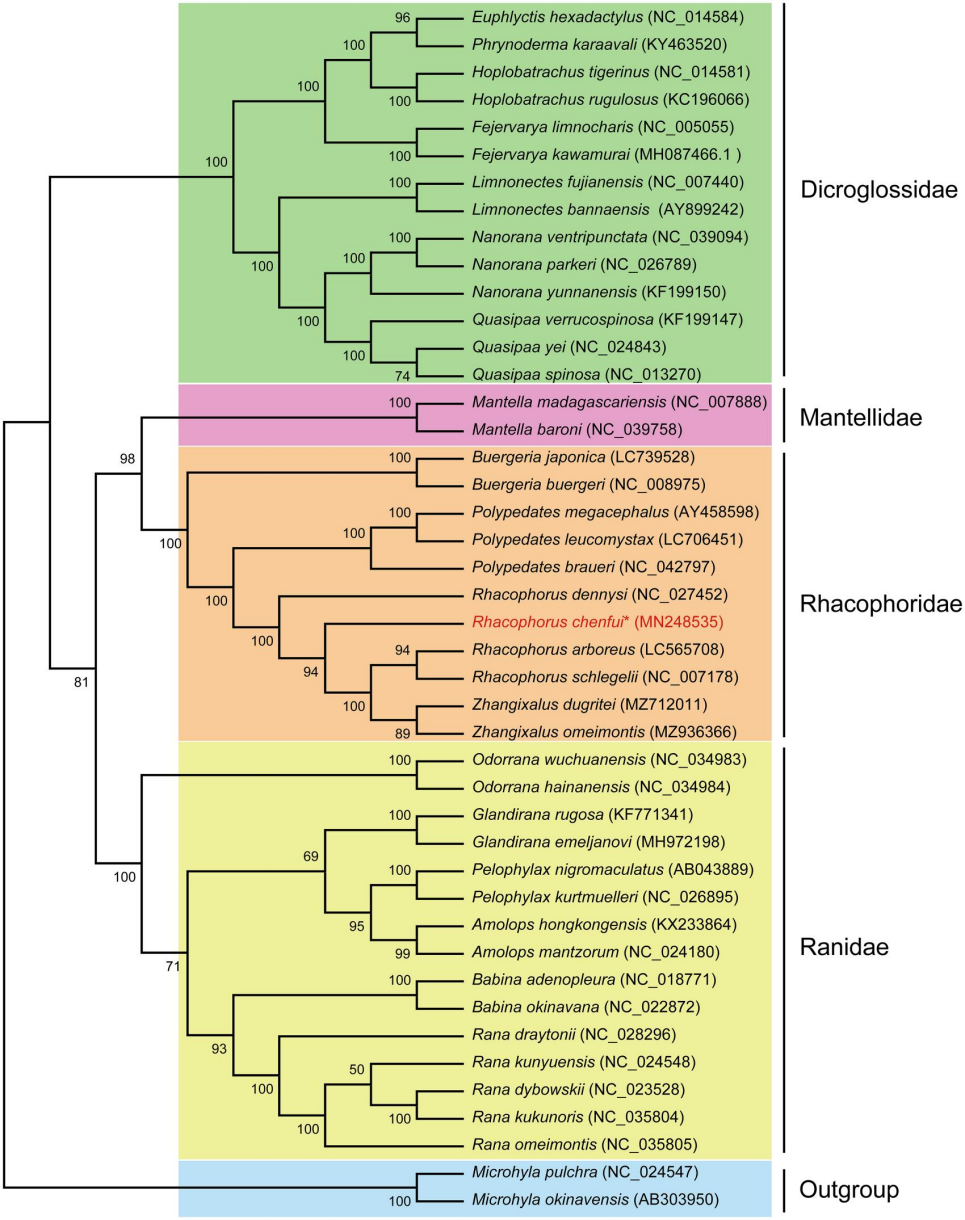
The phylogenetic tree inferred by the Maximum Likelihood (ML). Phylogenetic relationships of *Rhacophorus chenfui* and other 43 species based on 13 protein-coding genes (PCGs). *Microhyla pulahra* and *M. okinavensis* are used as an outgroup. GenBank accession numbers and bootstrap values of nodes are shown on the tree. The asterisks indicate new sequence generated in this study.

As shown in Figure 3 (Table S1), based on the complete mtDNA genome sequences of 44 species, the family Dicroglossidae, Ranidae, Mantellidae and Rhacophoridae are divided into four clades: 7 genera (*Quasipaa*, *Nanorana*, *Limnonectes*, *Fejervarya*, *Hoplobatrachus*, *Phynoderma* and *Euphlyctis*) form family Dicroglossidae, 1 genera (*Mantella*) form family Mantellidae, 4 genera (*Zhangixalus*, *Rhacophorus*, *Polypedates* and *Buergeria*) form family Rhacophoridae, while 6 genera (*Odorrana*, *Glandirana*, *Amolops*, *Babina*, *Rana* and *Pelophylax*) form family Ranidae, and two outgroups (*Microhyla pulahra* and *M. okinavensis*). ML tree showed that *R. chenfui* formed a large branch with *Z. omeimontis*, *Z. dugritei*, *R. arboreus* and *R. schlegelii* together (Figure 3). *R. chenfui* is located at the base of this branch, indicating a relatively primitive evolution. Notably, *Rhacophorus* is clustered with *Zhangixalus*, which indicates that the genus *Rhacophorus* is paraphyletic.

## 4. Discussion and conclusion

The gene arrangement and composition of the mitogenome of *R. chenfui* were found to be similar to other reported species of the genus *Rhacophorus* (Liu et al. 2016) and most other previously sequenced tree frogs (Huang et al. 2016; Inagaki et al. 2020; Pyron and Wiens 2011). In this species, we found three consecutive tRNA^Met^ genes, which is relatively rare in amphibian species. The gene arrangements and composition are similar to that of the putative ancestral mitogenome placement of *Buergeria buergeri* in family Rhacophoridae (Sano et al. 2005; Huang et al. 2016; Inagaki et al. 2020; Sano et al. 2005).

The ML tree supports that *Z. omeimontis*, *Z. dugritei*, *R. arboreus*, *R. schlegelii*, and *R. dennysi* constitute a sister-group mitogenome relationship with *Polypedates leucomystax*, *P. braueri* and *P. megacephalus*, then genus *Buergeria* species. The inclusion of more related taxa on future whole mitogenome phylogenetic analyses may help to understand the phylogeny of *Zhangixalus*, *Rhacophorus* and *Polyedates* (Chen et al. 2017; Li et al. 2008, 2021; Pyron and Wiens 2011). In conclusion, this study will provide important information for future taxonomic, biogeographical, systematic, and genetic studies of Rhacophoridae. Therefore, increasing the number of mitogenomes from species within the family Rhacophoridae will contribute to the taxonomic classification of this family. Our study presented the complete mitogenome sequence of *R. chenfui*, providing fundamental data for resolving phylogenetic and genetic issues related to the genus *Rhacophorus*. It also suggested that the relationships in the Rhacophoridae may need further investigation.

## Supplementary Material

Table S1.docx

Figure S2.jpg

Figrue S1.jpg

## Data Availability

The genome sequence data that support the findings of this study are openly available in GenBank of NCBI at https://www.ncbi.nlm.nih.gov/ under the accession no. MN248535. The associated ‘BioProject’, Bio-Sample, and SRA numbers are PRJNA1010143, SAMN37182129, and SRR25776678 respectively.

## References

[CIT0001] AmphibiaChina. 2023. The database of Chinese amphibians. Electronic Database accessible at http://www.amphibiachina.org/. Kunming Institute of Zoology (CAS), Kunming, Yunnan, China.

[CIT0002] Bernt M, Donath A, Jühling F, Externbrink F, Florentz C, Fritzsch G, Pütz J, Middendorf M, Stadler PF. 2013. MITOS: improved de novo metazoan mitochondrial genome annotation. Mol Phylogenet Evol. 69(2):313–319. doi:10.1016/j.ympev.2012.08.023. 22982435

[CIT0003] Boore JL. 2001. Complete mitochondrial genome sequence of the polychaete annelid *Platynereis dumerilii*. Mol Biol Evol. 18(7):1413–1416. doi:10.1093/oxfordjournals.molbev.a003925. 11420379

[CIT0004] Chen Z, Li H, Zhu Y, Feng Q, He Y, Chen X. 2017. Molecular phylogeny of the family Dicroglossidae (Amphibia: Anura) inferred from complete mitochondrial genomes. Biochem Syst Ecol. 71:1–9. doi:10.1016/j.bse.2017.01.006.

[CIT0005] Fei L, Ye CY, Jiang JP. 2012. Colored atlas of Chinese amphibians and their distributions. Chengdu (China): Sichuan Science and Technology Press.

[CIT0006] Frost DR. 2020. Amphibian Species of the World: an Online Reference. Version 6.0. New York (NY): American Museum of Natural History, Available at: http://research.amnh.org/herpetology/amphibia/index.html.

[CIT0007] Greiner S, Lehwark P, Bock R. 2019. OrganellarGenomeDRAW (OGDRAW) version 1.3.1: expanded toolkit for the graphical visualization of organellar genomes. Nucleic Acids Res. 47(W1):W59–W64. doi:10.1093/nar/gkz238. 30949694 PMC6602502

[CIT0008] Huang M, Duan R, Tang T, Zhu C, Wang Y. 2016. The complete mitochondrial genome of *Hyla tsinlingensis* (Anura: Hylidae). Mitochondrial DNA A DNA Mapp Seq Anal. 27(6):4130–4131. doi:10.3109/19401736.2014.1003877. 25629483

[CIT0009] Huang M, Lv T, Duan R, Zhang S, Li H. 2016. The complete mitochondrial genome of *Rhacophorus dennysi* (Anura: Rhacophoridae) and phylogenetic analysis. Mitochondrial DNA A DNA Mapp Seq Anal. 27(5):3719–3720. doi:10.3109/19401736.2015.1079873. 26329505

[CIT0010] Inagaki H, Haramoto Y, Kubota HY, Shigeri Y. 2020. Complete mitochondrial genome sequence of Japanese forest green tree frog (*Rhacophorus arboreus*). Mitochondrial DNA B Resour. 5(3):3347–3348. doi:10.1080/23802359.2020.1820396. 33458164 PMC7782537

[CIT0011] IUCN. 2023. *Zhangixalus omeimontis.* The IUCN Red List of Threatened Species. Version 2022-2. https://www.iucnredlist.org. Accessed on 20 August 2023.

[CIT0012] Jones DT, Taylor WR, Thornton JM. 1992. The rapid generation of mutation data matrices from protein sequences. Comput Appl Biosci. 8(3):275–282. 2. doi:10.1093/bioinformatics/8.3.275. 1633570

[CIT0013] Kumar S, Stecher G, Tamura K. 2016. Mega 7: molecular evolutionary genetics analysis version 7.0 for bigger datasets. Mol Biol Evol. 33(7):1870–1874. doi:10.1093/molbev/msw054. 27004904 PMC8210823

[CIT0014] Kurabayashi A, Sumida M. 2009. PCR primers for the Neobatrachian mitochondrial genome. Curr Herpetol. 28:1–11.

[CIT0015] Li J, Che J, Bain RH, Zhao E, Zhang Y. 2008. Molecular phylogeny of Rhacophoridae (Anura): a framework of taxonomic reassignment of species within the genera *Aquixalus*, *Chiromantis*, *Rhacophorus*, and *Philautus*. Mol Phylogenet Evol. 48(1):302–312. doi:10.1016/j.ympev.2008.03.023. 18442928

[CIT0016] Li Y, Zhang H, Wu X, Li D, Yan P, Wu X. 2021. The complete mitochondrial genome of *Rhacophorus dennysi* (Anura: Rhacophoridae) with novel gene arrangements and its phylogenetic implications. Pakistan J Zool. 53(6):2013–2019.

[CIT0017] Ojala D, Montoya J, Attardi G. 1981. tRNA punctuation model of RNA processing in human mitochondria. Nature. 290(5806):470–474. doi:10.1038/290470a0. 7219536

[CIT0018] Pyron RA, Wiens JJ. 2011. A large-scale phylogeny of Amphibia including over 2800 species, and a revised classification of extant frogs, salamanders, and caecilians. Mol Phylogenet Evol. 61(2):543–583. doi:10.1016/j.ympev.2011.06.012. 21723399

[CIT0019] Rowley JJL, Thuy LET, Tran D, Dao TA, Stuart BL, Huy HD. 2010. A new tree frog of the genus *Rhacophorus* (Anura: Rhacophoridae) from southern Vietnam. Zootaxa. 2727(1):45–55. doi:10.11646/zootaxa.2727.1.4.

[CIT0020] Sano N, Kurabayashi A, Fujii T, Yonekawa H, Sumida M. 2005. Complete nucleotide sequence of the mitochondrial genome of Schlegel’s tree frog *Rhacophorus schlegelii* (family Rhacophoridae): duplicated control regions and gene rearrangements. Genes Genet Syst. 80(3):213–224. doi:10.1266/ggs.80.213. 16172533

[CIT0021] Shan X, Xia Y, Kakehashi R, Kurabayashi A, Zou FD, Zeng XM. 2016. Complete mitochondrial genome of *Amolops mantzorum* (Anura: Ranidae). Mitochondrial DNA A DNA Mapp Seq Anal. 27(1):705–707. doi:10.3109/19401736.2014.913152. 24810067

[CIT0022] Stecher G, Tamura K, Kumar S. 2020. Molecular evolutionary genetics analysis (MEGA) for macOS. Mol Biol Evol. 37(4):1237–1239. doi:10.1093/molbev/msz312. 31904846 PMC7086165

[CIT0023] Tamura K, Stecher G, Kumar S. 2021. MEGA 11: molecular evolutionary genetics analysis version 11. Mol Biol Evol. 38(7):3022–3027. doi:10.1093/molbev/msab120. 33892491 PMC8233496

[CIT0024] Wang JJ, Yang MF, Dai RH, Li H, Wang XY. 2018. Characterization and phylogenetic implications of the complete mitochondrial genome of Idiocerinae (Hemiptera: Cicadellidae). Int J Biol Macromol. 120(Pt B):2366–2372. doi:10.1016/j.ijbiomac.2018.08.191. 30179694

[CIT0025] Xue R, Liu J, Yu J, Yang J. 2016. The complete mitogenome of Amolops loloensis and related phylogenetic relationship among Ranidae. Mitochondrial DNA A DNA Mapp Seq Anal. 27(6):4629–4630. doi:10.3109/19401736.2015.1101589. 26681370

[CIT0026] Zhang JY, Zhang LP, Yu DN, Storey KB, Zheng RQ. 2018. Complete mitochondrial genomes of *Nanorana taihangnica* and *N. yunnanensis* (Anura: Dicroglossidae) with novel gene arrangements and phylogenetic relationship of Dicroglossidae. BMC Evol Biol. 18(1):26. doi:10.1186/s12862-018-1140-2. 29486721 PMC6389187

