# Supplementary material for: The complete mitochondrial genome of the Rhacophorus chenfui Liu, 1945 and its phylogenetic analyses
Source: Mitochondrial DNA B Resour. 2024 Nov 12;9(11):1522–7. doi: 10.1080/23802359.2024.2427829 (PMC11559012; doi:10.1080/23802359.2024.2427829)
Supplement: Table S1.docx [file TMDN_A_2427829_SM5965.docx]

**Table S1. Species mentioned in the present study with their GenBank accession number and references.**

| **No.** | **Superfamily** | **Family** | **Genus** | **Species** | **length (bp)** | **Accession no.** | **References** |
| --- | --- | --- | --- | --- | --- | --- | --- |
| 1 | Ranoidea | Rhacophoridae | *Rhacophorus* | *Rhacophorus chenfui* | 20,520 | MN248535 | **This study** |
| 2 | Ranoidea | Rhacophoridae | *Rhacophorus* | *Rhacophorus arboreus* | 22,236 | LC565708 | Inagaki H, et al, 2020 |
| 3 | Ranoidea | Rhacophoridae | *Rhacophorus* | *Rhacophorus dennysi* | 18,052 | NC_027452 | Wu X, et al, 2015 |
| 4 | Ranoidea | Rhacophoridae | *Rhacophorus* | *Rhacophorus schlegelii* | 21,359 | NC_007178 | Sano N, et al, 2005 |
| 5 | Ranoidea | Rhacophoridae | *Zhangixalus* | *Zhangixalus omeimontis* | 19,782 | MZ936366 | unpublished |
| 6 | Ranoidea | Rhacophoridae | *Zhangixalus* | *Zhangixalus dugritei* | 19,412 | MZ712011 | unpublished |
| 7 | Ranoidea | Rhacophoridae | *Polypedates* | *Polypedates leucomystax* | 20,794 | LC706451 | unpublished |
| 8 | Ranoidea | Rhacophoridae | *Polypedates* | *Polypedates megacephalus* | 16,473 | AY458598 | Zhang P, et al, 2005 |
| 9 | Ranoidea | Rhacophoridae | *Polypedates.* | *Polypedates braueri* | 19,904 | NC_042797 | Huang A. et al, 2019 |
| 10 | Ranoidea | Rhacophoridae | *Buergeria* | *Buergeria buergeri* | 19,959 | NC_008975 | Sano N, et al, 2004 |
| 11 | Ranoidea | Rhacophoridae | *Buergeria* | *Buergeria japonica* | 22,274 | LC739528 | Asaeda Y, et al, 2023 |
| 12 | Ranoidea | Mantellidae | *Mantella* | *Mantella baroni* | 20,945 | NC_039758 | Richard Y, et al, 2018 |
| 13 | Ranoidea | Mantellidae | *Mantella* | *Mantella madagascariensis* | 22,874 | NC_007888 | Kurabayashi A, et al, 2006 |
| 14 | Ranoidea | Dicroglossidae | *Euphlyctis* | *Euphlyctis hexadactylus* | 20,280 | NC_014584 | Alam M.S, et al, 2010 |
| 15 | Ranoidea | Dicroglossidae | *Euphlyctis* | *Euphlyctis karaavali* | 15,505 | KY463520 | Anoop V S, et al, 2017 |
| 16 | Ranoidea | Dicroglossidae | *Fejervarya* | *Fejervarya limnocharis* | 17,717 | NC_005055 | Liu Z, et al, 2005 |
| 17 | Ranoidea | Dicroglossidae | *Fejervarya* | *Fejervarya kawamurai* | 17,650 | MH087466 | Cheng J, et al, 2018 |
| 18 | Ranoidea | Dicroglossidae | *Hoplobatrachus* | *Hoplobatrachus tigerinus* | 20,462 | NC_014581 | Alam M.S, et al, 2010 |
| 19 | Ranoidea | Dicroglossidae | *Hoplobatrachus* | *Hoplobatrachus rugulosus* | 16,903 | KC196066 | unpublished |
| 20 | Ranoidea | Dicroglossidae | *Limnonectes* | *Limnonectes bannaensis* | 16,867 | AY899242 | Zhang J F, et al, 2009 |
| 21 | Ranoidea | Dicroglossidae | *Limnonectes* | *Limnonectes fujianensis* | 18,293 | NC_007440 | unpublished |
| 22 | Ranoidea | Dicroglossidae | *Nanorana* | *Nanorana parkeri* | 17,837 | NC_026789 | Jiang L, et al, 2015 |
| 23 | Ranoidea | Dicroglossidae | *Nanorana* | *Nanorana ventripunctata* | 18,373 | NC_039094 | Jiang L, et al, 2018 |
| 24 | Ranoidea | Dicroglossidae | *Nanorana* | *Nanorana yunnanensis* | 23,685 | KF199150 | Zhang J-Y, et al, 2018 |
| 25 | Ranoidea | Dicroglossidae | *Quasipaa* | *Quasipaa yei* | 17,072 | NC_024843 | Chen Z, et al, 2014 |
| 26 | Ranoidea | Dicroglossidae | *Quasipaa* | *Quasipaa spinosa* | 18,012 | NC_013270 | Zhou Y, et al, 2009 |
| 27 | Ranoidea | Dicroglossidae | *Quasipaa* | *Quasipaa verrucospinosa* | 15,063 | KF199147 | unpublished |
| 28 | Ranoidea | Ranidae | *Rana* | *Rana kunyuensis* | 22,255 | NC_024548 | Li J, et al, 2014 |
| 29 | Ranoidea | Ranidae | *Rana* | *Rana dybowskii* | 18,864 | NC_023528 | Li J, et al, 2014 |
| 30 | Ranoidea | Ranidae | *Rana* | *Rana draytonii* | 17,805 | NC_028296 | unpublished |
| 31 | Ranoidea | Ranidae | *Rana* | *Rana omeimontis* | 19,934 | NC_035805 | unpublished |
| 32 | Ranoidea | Ranidae | *Rana* | *Rana kukunoris* | 18,863 | NC_035804 | unpublished |
| 33 | Ranoidea | Ranidae | *Babina* | *Babina adenopleura* | 18,982 | NC_018771 | Yu D N, et al, 2012 |
| 34 | Ranoidea | Ranidae | *Babina* | *Babina okinavana* | 19,959 | NC_022872 | Kakehashi R, et al, 2013 |
| 35 | Ranoidea | Ranidae | *Amolops* | *Amolops hongkongensis* | 14,798 | KX233864 | Zhang J Y, et al, 2018 |
| 36 | Ranoidea | Ranidae | *Amolops* | *Amolops mantzorum* | 17,744 | NC_024180 | Shan X, et al, 2014 |
| 37 | Ranoidea | Ranidae | *Odorrana* | *Odorrana wuchuanensis* | 18,256 | NC_034983 | Huang Y, et al, 2016 |
| 38 | Ranoidea | Ranidae | *Odorrana* | *Odorrana hainanensis* | 17,986 | NC_034984 | Huang Y, et al, 2017 |
| 39 | Ranoidea | Ranidae | *Pelophylax* | *Pelophylax nigromaculatus* | 17,804 | AB043889 | Sumida M, et al, 2001 |
| 40 | Ranoidea | Ranidae | *Pelophylax* | *Pelophylax kurtmuelleri* | 18,020 | NC_026895 | Hofman S, et al, 2015 |
| 41 | Ranoidea | Ranidae | *Glandirana* | *Glandirana rugosa* | 17,426 | KF771341 | Xia Y, et al, 2014 |
| 42 | Ranoidea | Ranidae | *Glandirana* | *Glandirana emeljanovi* | 17,668 | MH972198 | Eo SH, et al, 2019 |
| 43 | Microhyloidea | Microhylidae | *Microhyla* | *Microhyla okinavensis* | 16,717 | AB303950 | Igawa T, et al, 2008 |
| 44 | Microhyloidea | Microhylidae | *Microhyla* | *Microhyla pulchra* | 16,744 | NC_024547 | Wu X, et al, 2014 |
