# Supplementary figures and images for: The complete mitochondrial genome of the Rhacophorus chenfui Liu, 1945 and its phylogenetic analyses
Source: Mitochondrial DNA B Resour. 2024 Nov 12;9(11):1522–7. doi: 10.1080/23802359.2024.2427829 (PMC11559012; doi:10.1080/23802359.2024.2427829)

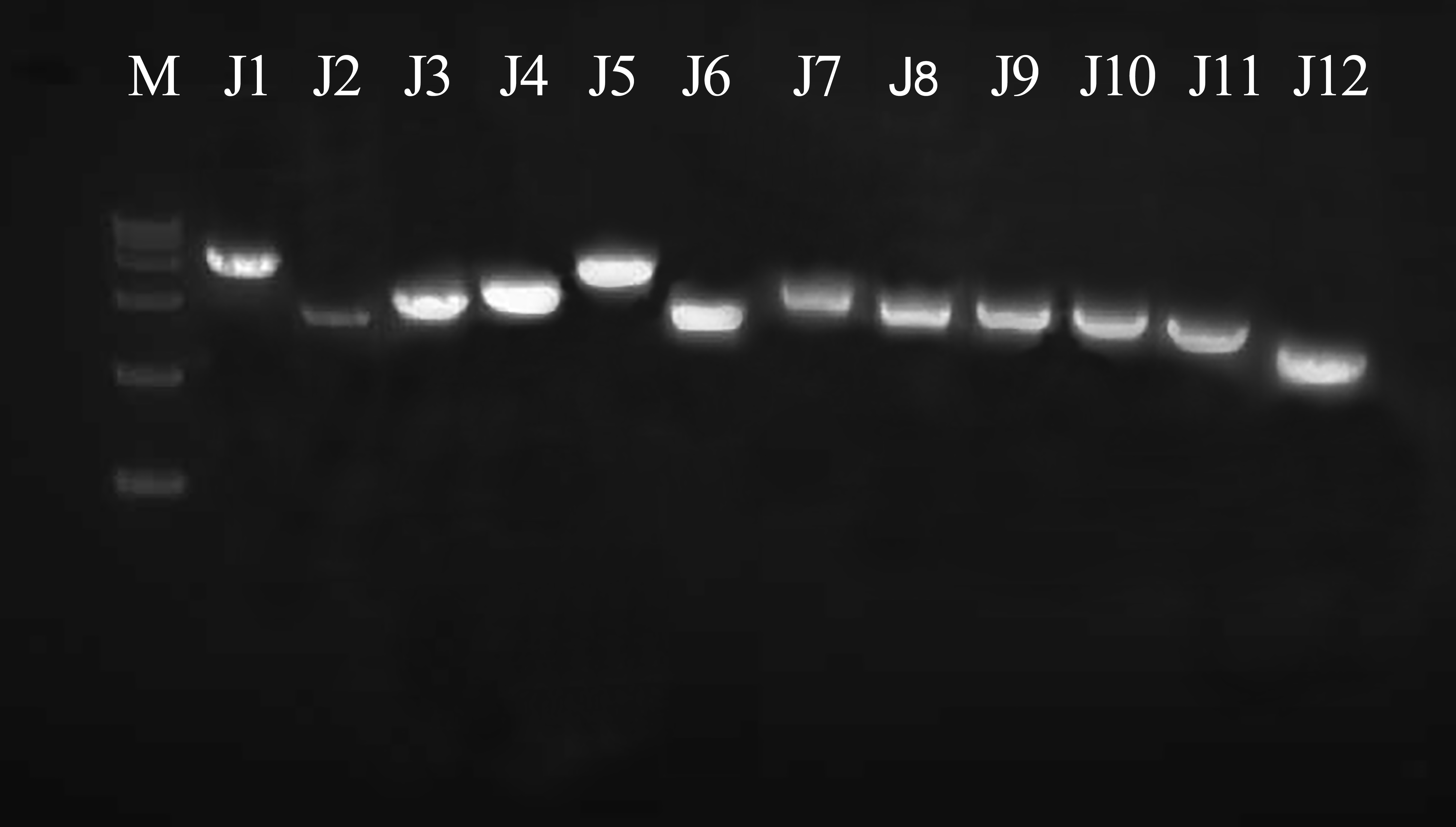

Supplement: Figure S2.jpg [file TMDN_A_2427829_SM5964.jpg]

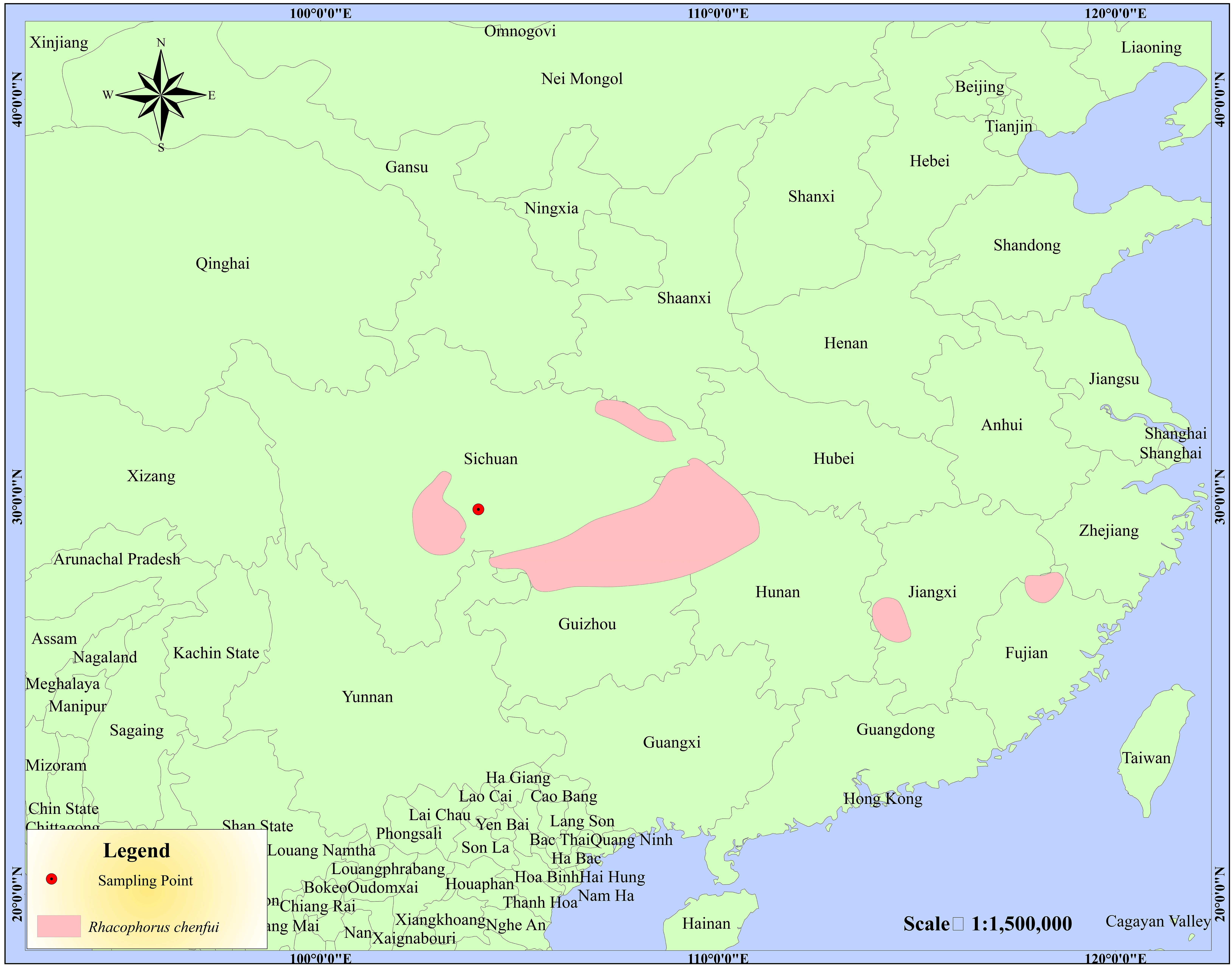

Supplement: Figrue S1.jpg [file TMDN_A_2427829_SM5963.jpg]
